# Supplementary material for: CD90+ liver cancer cells modulate endothelial cell phenotype through the release of exosomes containing H19 lncRNA
Source: Mol Cancer. 2015 Aug 14;14:155. doi: 10.1186/s12943-015-0426-x (PMC4536801; doi:10.1186/s12943-015-0426-x)
Supplement: Additional file 1: — (a) Characterization of isolated exosomes. Left panel: DSL for exosomes released by SKHep Middle panel: Western blot forTsg101 and HSC70 in SkHep cells and their relative exosomes. Right panel: Confocal microscopy analysis on HUVECs treated for 1, 3 and 6 hours with 5 mg/ml of SKHep-derived exosomes. HUVECs were stained with phalloidin Alexa Fluor (green), nuclear counterstaining was performed using DAPI (blue), exosomes were labelled with PKH26 (red). (b) Target analysis. Real time-PCR analysis on HUVECs treated for 18 h with 5 mg/ml of SkHep-derived exosomes. Normalized for b-actin the DDct were indicated as fold of induction respect to control (untreated cells). *p<0.05. (c) Tubulogenesis of HUVECs after exosomes treatment. Matrigel assay performed on HUVECs cells after 18 hour of 5 mg/ml SkHep-derived exosomes. Left panel: phase contrast, magnification 20x. Right panel: quantification of matrigel assay expresses as length of cable as arbitrary unit **p<0.01. (d) Adhesion assay of SkHep cells on HUVECs. Left panel: phase contrast, magnification 10X. Right Panel: quantification of Huh7 or SKHep cells adherent on HUVECs, performed by counting the number of CD90+adherent cells (violet) per field ***p<0.001. (e) Analysis on H19 expression in exosomes. Real time-PCR analysis for H19 expression performed on SkHep-derived exosomes respect to Huh7 derived exosomes. Normalized for b-actin the DDct were indicated as fold of induction. **p<0.01 (f) Comparison of H19 expression in HUVECs after exosomes treatment. Real time-PCR analysis for H19 expression on HUVECs treated for 18 h with 5 mg/ml of SkHep or Huh7 exosomes. Normalized for b-actin the DDct were indicated as fold of induction respect to control (untreated cells). (DOCX 855 kb) [file 12943_2015_426_MOESM1_ESM.docx]

CD90+ Liver Cancer Cells Modulate Endothelial Cell Phenotype through the Release of Exosomes Containing H19 lncRNA

Alice Conigliaro[1*#], Viviana Costa[2*], Alessia Lo Dico[3*], Laura Saieva[3], Simona Buccheri[3-4], Francesco Dieli[3], Mauro Manno[5], Samuele Raccosta[5], Carmine Mancone[1,6], Marco Tripodi[6,7], Giacomo De Leo[3], Riccardo Alessandro[3,8#]

*** These authors contributed equally to this work**

1. Dipartimento di Biotecnologie cellulari ed Ematologia, Sapienza University of Rome
2. Laboratory of Tissue Engineering - Innovative Technology Platforms for Tissue Engineering (PON01-00829), Rizzoli Orthopedic Institute, Palermo, Italy;
3. Dipartimento di Biopatologia e Biotecnologie Mediche, University of Palermo, Italy;
4. Servizio di Diabetologia, Dipartimento per la cura e lo studio della patologie addominali e dei trapianti addominali. ISMETT IRCCS. Palermo, Italy
5. Institute of Biophysics, National Research Council of Italy, Palermo, Italy;
6. National Institute for Infectious Diseases L. Spallanzani, IRCCS, Rome, Italy.
7. Istituto Pasteur-Fondazione Cenci Bolognetti, Dipartimento di Biotecnologie Cellulari ed Ematologia, Sapienza University of Rome.
8. Institute of Biomedicine and Molecular Immunology (IBIM), National Research Council of Italy, Palermo, Italy.

# Corresponding authors:

Alice Conigliaro, PhD. Dipartimento di Biotecnologie Cellulari ed Ematologia, c/o Policlinico Umberto I, V Clinica Medica Viale Regina Elena, 324-00161 Rome, Italy E-mail: alice.conigliaro@uniroma1.it Phone: +39-06-445 37 31 Fax: +39-06-446 28 91

Riccardo Alessandro: Full Professor of Biology and Genetics. Dipartimento di Biopatologia e Biotecnologie Mediche. University of Palermo, Via Divisi 83-90133 Palermo, Italy

E-mail: riccardo.alessandro@unipa.it Phone: +39-091-655 46 08 Fax: +39-091-655 46 24

Electronic word count: 5809

Number of figures: 5.

Abbreviations: lncRNA: long-non-coding RNA; HCC: hepatocellular carcinoma; HUVEC: human umbilical vein endothelial cell; CSC: cancer stem cell; VEGF: vascular endothelial growth factor; ICAM-1: intercellular adhesion molecule-1

Keywords: Exosomes, Long-non-coding RNA H19, CD90+ liver cancer cells, angiogenesis.

The authors have no conflict of interests to declare.

Financial supports: MIUR Ministero dell'Universita e Ricerca Scientifica (FIRB 2012-RBFR12NSCF_002); AIRC Associazione Italiana per la Ricerca sul Cancro (12763)

Author's contributions: AC, RA, VC, ALD contributed to the conception and design of the study. AC, RA, VC, ALD, LS, SB, SR, MM, RA contributed to the generation, collection, assembly, analysis and/or interpretation of data. AC, RA, VC, ALD, MT, CM, FD, GDL, contributed to drafting or revision of the manuscript. All authors approved the final version of the manuscript.

Abstract

Background: **CD90+ liver cancer cells have been described as cancer stem-cell-like, displaying aggressive and metastatic phenotype. Using two different *in vitro* models, already described as CD90+ liver cancer stem cells, our aim was to study their interaction with endothelial cells mediated by the release of exosomes.** ***Methods:*** **Exosomes were isolated and characterized from both liver CD90+ cells and hepatoma cell lines. Endothelial cells were treated with exosomes, as well as transfected with a plasmid containing the full length sequence of the long non-coding RNA (lncRNA) H19. Molecular and functional analyses were done to characterize the endothelial phenotype after treatments.** ***Results:*** **Exosomes released by CD90+ cancer cells, but not by parental hepatoma cells, modulated endothelial cells, promoting angiogenic phenotype and cell-to-cell adhesion. LncRNA profiling revealed that CD90+ cells were enriched in lncRNA H19, and released this through exosomes. Experiments of gain and loss of function of H19 showed that this LncRNA plays an important role in the exosome-mediated phenotype of endothelial cells.** ***Conclusions:*** **Our data indicate a new exosome-mediated mechanism by which CSC-like CD90+ cells could influence their tumor microenvironment by promoting angiogenesis. Moreover, we suggest the lncRNA H19 as a putative therapeutic target in hepatocellular carcinoma.**

Background

Hepatocellular carcinoma (HCC) is the third leading cause of cancer mortality worldwide [1]. Primary HCC lesions can be removed completely when detected at an early stage, but intrahepatic recurrence of HCC and extrahepatic metastasis are very frequent, giving rise to a poor prognosis for patients [2, 3]. It is widely accepted that both differentiated hepatocytes and cells with progenitor characteristics, known

as cancer stem cells (CSCs), can cause HCC [4-7]. Forty percent of HCCs are clonal, and potentially derived from progenitor/stem cells. Moreover, these cells have a critical role in the development and progression of HCC [8]. Liver CSCs have been isolated from primary HCC specimens and patients' sera as circulating cells, and from HCC cell lines by use of surface antigens [9-11]. CD90, the epithelial cell adhesion molecule (EpCAM) and CD133 have been found to recognize three distinct cell populations that differ from one another in features and behavior in determining cancer phenotypes [12].

CD90 (Thy-1) is a 25-37 kDa glycophosphatidylinositol (GPI)-anchored protein expressed by several cells such as T-cells, neurons, endothelial cells and fibroblasts. It is involved in cell-to-cell and cell-matrix interaction, apoptosis, adhesion, migration, fibrosis, and cancer development [13]. Concerning the liver, the expression of CD90 has been linked to hepatic stem/progenitor cells [14] and, during tumor growth, it has been correlated with an aggressive phenotype [15], and associated with low differentiated HCC and poor prognosis [16-18]. CD90+ CSCs obtained from HCC cell lines, from tumor tissues and peripheral blood as circulating cancer cells displayed, in contrast to the other CSC populations, a mesenchymal phenotype and, most importantly, a greater capacity to metastasize when injected into immunodeficient mice [19, 11, 12]. Moreover, recent data associate CD90 expression with early HCC recurrence [20]. Gene expression and miRNA analysis in CD90+ HepG2 cells have revealed an imbalance in the expression of apoptosis and anti-apoptosis genes compared with CD90 negative cells [21]. However, we are still far from understanding the molecular mechanisms underlying the more aggressive and metastatic phenotype of these cells compared with the other liver cancer cells.

Tumor development is dependent on the reciprocal interactions between cancer cells and the surrounding microenvironment. It is well known that in addition to pathways involving cell-to-cell contact and the release of soluble factors, cancer cells are able to communicate with the tumor

microenvironment (e.g., myeloid cells, fibroblasts, endothelial cells) through the intercellular exchange of proteins and genetic materials via exosomes [22].

Exosomes are spherical membrane vesicles of endocytic origin, with an average size of 40 to 150 nm [23], released by both normal and diseased cells after the fusion of multivesicular bodies with the plasma membrane. First considered as collectors of cellular waste materials, exosomes have assumed a leading role in the regulation of the tumor microenvironment. Depending on their content, exosomes can affect tumor cells and surrounding stroma by influencing major cellular pathways, such as apoptosis, cell differentiation, angiogenesis, and metastasis [24]. These vesicles act as cargos that release bioactive molecules e.g., lipids, proteins, and nucleic acids in target cells. Interestingly, recent observations have identified a vesicle–mediated transfer of lncRNAs as an important mechanism in the development of HCC [25]. In this paper we demonstrate that CD90+ cells, derived from HCC cell lines, release exosomes that, in turn, are able to influence endothelial cells by promoting angiogenesis and stimulating their adhesive properties. Furthermore, our results suggest the lncRNA H19 as a possible mediator of angiogenic effects.

Results

CD90+ cells show a mesenchymal phenotype and actively release exosomes.

As reported by Yang and colleagues [11], highly positive CSC-like CD90+ cells were isolated by cell sorting, starting from Huh7 cell line presenting a mean of 4% CD90+ cells and 2% CD90 high-expressing cells. After sorting, the purity of the selected CD90+ population was monitored during cell passages by FACS analysis, and the cells were kept in culture until they maintained a positivity for CD90 of over 90% (at approximately the 40th passage). Isolated CD90+ cells, in contrast to the parental Huh7 and as already described by others [12], showed a mesenchymal phenotype, revealing a

delocalized E-Cadherin and a lack of expression of HNF4α, a master regulator of hepatocytic differentiation (Figure 1A). On the contrary, most of the cells were positive for vimentin, a component of intermediate filaments in mesenchymal cells (Figure 1A). In order to evaluate the ability of Huh7 and its CD90+ subpopulation to release nanovesicles, the conditioned medium was collected, and the vesicles isolated as described by members of our group [26, 27]. Measures obtained by DLS revealed, in the ultracentrifuged cell culture medium, vesicles with an average size in diameter of 50nm and 100nm from CD90+ or Huh7 cell medium, respectively (Figure 1B). This in line with the exosomes dimensions between 30 and 150 nm [28]. Moreover, Western blot analyses showed that Alix and Tsg101 markers are expressed but not enriched in exosomes released by CD90+Huh7 (Figure 1C).

Exosomes released by CD90+ cells affect HUVECs by promoting tube formation and cell-cell adhesion.

CD90+CSCs have been associated with metastasis and early recurrence in HCC [20, 12]. In order to evaluate whether the CD90+ cells were able to influence the tumor microenvironment, we treated HUVECs with exosomes released by CD90+ Huh7 cells or Huh7 parental cells (CD90+exo and Huh7exo). Endothelial cells rapidly internalized exosomes from both cell types; uptake was evident after one-hour incubation at 37°C, and increased over the course of six hours (Figure 1D). Eighteen hours after exosome treatment, real-time PCR analysis revealed that the addition of CD90+exo, but not of Huh7exo, highly increased the mRNA levels of the pro-angiogenic factor VEGF and its receptor VEGF-R1 in endothelial cells (Figure 2A). ELISA assay showed that HUVECs treated with CD90+exo released three-fold more VEGF (Figure 2B, left panel). Moreover, a significant increase in the number and the length of tubular-like structures was observed when HUVECs were treated with CD90+exo compared with Huh7exo (Figure 2B, middle and right panels).

Liver CD90+ CSCs were found circulating in HCC patients and in metastatic colonies [19]. For this reason, we tested the ability of exosomes released by the hepatoma cell line or by sorted CD90+ cells to modulate the adhesion to an endothelial cell monolayer, a crucial event for intra- or extra-vasation. As revealed by real-time PCR (Figure 2A), and confirmed by FACS analysis (Figure 2C), treatment of HUVECs with CD90+-derived exosomes modulated intercellular adhesion molecules, inducing an increase in the expression of ICAM-1. No significant differences were found in VCAM and VE-Cadherin gene expression (data not shown). To validate our data, we did an adhesion assay, pre-treating endothelial cells with exosomes. As shown in Figure 2D, CD90+exo caused a two-fold increase in adhering cells compared with pre-treatment Huh7-exo.

To further confirm our observation, the same experiments were performed with SkHep, a hepatoma cell line already characterized as 100% CD90+, and displaying mesenchymal stem cell characteristics [29]. Supplementary Figure 1A illustrates the characterization of exosomes released by SkHep and their uptake by HUVECs, that present different features compared to CD90+ Huh7 derived exosomes. In addition, measures obtained by DLS revealed, in the ultracentrifuged SKHep culture medium, exosomes with an average size in diameter of 70nm expressing high level of the exosomal markers TSG101 and HSC70. As observed for CD90+exo, the SkHep-derived exosomes induced a pro-angiogenic stimulus in endothelial cells, modifying their transcriptional profile and enhancing tube formation in matrigel, as well as increasing the adhesive properties of HUVECs (Supplementary Figure 1 from B to D).

In summary, our results showed that exosomes released by CSC-like CD90+ liver cells, but not from hepatoma cells, induce pro-angiogenic stimuli in HUVECs, and influence the adhesion between CD90+ cells and endothelial cells.

CD90+ cells express the lncRNA H19 and release it via exosomes.

It has been confirmed that dysregulation of lncRNAs is associated with several human tumors and, recently, a contribution of lncRNAs to hepatocarcinogenesis was found [30-32]. In order to clarify the molecular mechanism driving the modifications induced in HUVECs by CD90+-derived exosomes we did an lncRNA profile study in CD90+ cells and parental Huh7 by analyzing the expression of 90 different lncRNAs. In Figure 3A (left and middle panel) , the RNAs over-expressed in CD90+ cells compared with Huh7 parental cells with at least a ten-fold increase are listed. Among these, Air, Hotair, LincRNA-ROR, Hulc, and H19 have already been identified as positively correlated with hepatocellular carcinoma [31, 33, 34]. We focused our interest on H19, expression of which has been previously associated with metastasis [35, 36]. In line with recent articles, which have demonstrated that hepatocellular carcinoma cells release exosomes containing lncRNA [25, 37], we investigated the expression in exosomes, of those LncRNAs that we found overexpressed in cells. As shown in figure 3A right panel the LncProfiler performed on CD90+ Huh7 and Huh7-derived exosomes evidences that H19 was 10-fold up-regulated in exosomes derived from CD90+ Huh7, compared to parental cell line. The real time PCR confirmed that vesicles released by CD90+cells (both sorted or SkHep cells) are highly enriched in H19 transcript compared with vesicles from Huh7 parental cells (Figure 3B, S1E). Moreover, treatment with CD90+-derived exosomes induced in HUVECs an increase in H19 transcript (Figure 3C, S1F). These data suggest a transport of H19 lncRNA from CD90+ cells to HUVECs, even if we cannot exclude a stimulation of endogenous lncRNA.

LncRNA H19 stimulates angiogenesis and promotes the adhesion of CD90+ cells to endothelial cell monolayer.

To investigate a possible role of H19 as mediator of pro-angiogenic and adhesive stimuli in HUVECs, we transfected endothelial cells with the entire sequence of the lncRNA H19 (pH19). As shown in Figure 4A, H19 overexpression in HUVECs induced a transcriptional modulation similar to that obtained after CD90+exo treatment. Real-time PCR indicated that the over expression of H19 induced a significant increase in the VEGF and ICAM1 transcripts, while, no modulation compared to controls was observed for the transcription of VEGF-R1, VCAM and VE-cadherin (Figure 4A right panel). The ELISA assay (Figure 4A left panel) found, for the first time to our knowledge, a substantial increase in VEGF release induced by lncH19. Moreover, a rise in the number and length of tubes was found in HUVECs transfected with pH19 (Figure 4B), while FACS analysis (Figure 4C) indicated an increase in the number of ICAM-1-expressing cells induced by H19 overexpression, thus explaining the more adhesive phenotype of HUVECs. The adhesion assay, in fact, revealed a two-fold increase in adhering CD90+ cells when HUVECs were transfected with pH19 (Figure 4D).

Overall, these data demonstrate, for the first time to our knowledge, the ability of the lncRNA H19 to stimulate angiogenesis, and to favor cell-cell interaction, allowing us to postulate H19 as a possible mediator of pro-metastatic properties of exosomes released by CD90+ cells. To confirm our hypothesis, lncH19 was silenced in HUVECs concomitantly with CD90+exo treatment. As shown in Figure 5A, the silencing of H19 abrogated the exosome-mediated induction of VEGFR1, while no modulation was revealed in the expression of ICAM1. Concerning the VEGF, even if the reduction of transcript did not appear significant (5A), the release of VEGF protein induced by exosome treatment was totally inhibited by H19 silencing (5B).

Discussion

CD90+ liver CSCs have been found in primary tumors, and circulating in the blood of HCC patients, and are associated with early recurrence, metastasis, and poor prognosis [19, 20]. Our study highlights the ability of CSC-like CD90+ cells, but not hepatoma cells, to influence endothelial cell phenotype through the release of exosomes.

In a solid tumor, the CSC's niche is composed of an extracellular matrix (ECM), mesenchymal stem cells, tumoral cells, immune cells, and endothelial cells, all of which converge in determining the fate of CSCs through extracellular signals [38]. Little is known about the modulation of the tumor microenvironment by CSCs. Several studies have described exosomes as signaling extracellular organelles that modulate the tumor microenvironment, promoting angiogenesis and tumor progression [27, 39]. Our data indicate that exosomes released by CSC-like CD90+ liver cells are able to promote an angiogenic phenotype in cultured endothelial cells. CD90+-derived exosomes induced in HUVECs an increase in the production and secretion of VEGF, the most powerful pro-angiogenic cytokine, as well as of its receptor VEGF-R1. This increase was accompanied by an amplification in the number and length of tube-like structures formed by HUVECs in culture.

It is abundantly documented that metastatic processes induce changes in the endothelial surface antigens, with an increase in adhesion molecules, which, in turn, favor the adhesion and the consequent intra- or extravasation of metastatic cells. We found that exosomes released by CD90+ cells, and not by hepatoma cells, increased the number of HUVECs expressing ICAM-1 and, more extensively, increased the adhesion between endothelial cells and the CSC-like CD90+ cells. Our data also indicate that the CD90+ released exosomes may be able to promote metastasis.

Recently, Patel et al. demonstrated that lncRNA could be selectively packaged in extracellular vesicles released by hepatoma cell lines and transported to other cells, with subsequent modulation of cellular

function [25, 40]. LncRNA are emerging as molecular players in several biological processes acting at epigenetic, transcriptional and post-transcriptional levels or processing small non-coding RNAs [41]. H19 was among the first lncRNAs to be identified and studied principally for its monallelic expression, and as regulator of IGF2 abundance [42, 43]. As already described for other lncRNAs, H19 can work as a microRNA sponge, miRNAs precursor, or epigenetic modulator [44, 45], and has been found overexpressed in several tumors, and able to promote tumor growth [46, 47] and progression [47, 35, 36]. Concerning the liver, H19 has been clearly involved in hepatocarcinogenesis [48] and hepatic metastases [49]. Several indications correlate H19 with angiogenesis [50, 51]. Northern analysis has indicated a high expression of H19 during development of rat aorta that decreases in differentiated tissue and, interestingly, re-appears following vascular injury *in vivo* and *in vitro* [50], though no observations of the overexpression of H19 in endothelial cells have been published.

In this study, we demonstrate, for the first time to our knowledge, that H19 is highly expressed in a subpopulation of hepatoma cells that expose the surface antigen CD90 and are characterized, by others, as CSC-like cells [29, 15, 12, 11]. We found that CD90+ cells package lncRNA H19 inside exosomes, thus delivering it to possible target cells. Exosomes released by CD90+ liver cancer cells could be internalized by endothelial cells, influencing these in a pro-metastatic way. Moreover, we identified in H19 an important player of this process. H19 overexpression in endothelial cells is able to up-regulate the VEGF production and release, increase the ability of HUVEC cells to arrange *in vitro* tubular-like structures, and promote heterotypic adhesion between endothelial cells and CSC-like liver cells. Silencing experiments revealed LncRNAH19 as the principal player of the exosome-mediated VEGF increase, while suggested the presence of other molecular actors that, transported or induced by CD90+-derived exosomes, and together with H19, affect endothelial cells in a pro-metastatic way. However, the mechanisms of action through which this lncRNA controls an endothelial phenotype

remain to be elucidated.

Conclusion

Our *in vitro* experiments demonstrated that CD90+ liver cancer cells release exosomes that, in turn, are able to affect endothelial cells in a pro-metastatic way. Exosomes derived by CD90+ cells and H19 may represent two new therapeutic targets for reducing recurrence and metastasis of HCC.

Material and Methods

Cell culture and reagents

Human umbilical vein endothelial cells (HUVECs) were obtained from Lonza (Verviers, Belgium) and grown in endothelial growth medium (EGM, bullet kit, Lonza) according to supplier's instructions. Huh7 cells and Sk-Hep cells were cultured in DMEM medium (Euroclone, UK), and supplemented with 10% fetal bovine serum (Euroclone, UK), 2 mM L-glutamine, 100 U/ml penicillin and 100 mg/ml streptomycin (Euroclone, UK).

Sorting Huh CD90+ cells

HuH-7 human hepatocellular carcinoma cells were stained with anti-CD90 PE (BD Pharmingen™ 555596), and surface marker was determined by flow cytometry. CD90+ and CD90- cells were sorted through a FACSAria I (BD Biosciences). A purity check was done after the sorting by re-running a small fraction of the sorted populations. All cells showed over 85% purity.

Immunocytochemistry

Immunocytochemistry was done on PFA 4% fixed cells, and stained with the following antibodies: the primary antibodies were anti-E-Cadherin (BD Biosciences 610181), anti-HNF4a (Abcam ab41898), and anti-Vimentin (Epitomics, 2707-1); the secondary antibodies were Alexa-Fluor 488 and Alexa-

Fluor 594, from Molecular Probes. The nuclei were stained with NucRed® Live 647 (Catalog number: R37106, Life Technologies), and preparations were analyzed by confocal microscopy (Leica TSC SP8).

Exosome preparation and characterization

Huh7, CD90+ Huh7 and Sk-Hep cells were grown with 10% ultracentrifugated FBS, and conditioned medium was collected 48 hours after culture; exosomes were subsequently isolated by serial centrifugation [26]. Briefly, culture medium was centrifuged subsequently for 5 min at 300 × g, 15 min at 3,000 × g, 30 minutes at 10,000 × g and ultracentrifuged 90 min at 100,000 × g in a Type 70 Ti, fixed angle rotor. Peletted exosomes were washed and then resuspended in PBS. Exosome protein content was determined with the Bradford assay (Pierce, Rockford, IL, USA). On average we recovered 10 micrograms of vesicles from 25 ml of conditioned medium from 3×10^6 cells. The intensity autocorrelation functions of diluted vesicle samples were measured by dynamic light scattering (DLS) using a Brookhaven Instruments BI-9000 correlator and a BI200-SM goniometer, equipped with a solid-state laser tuned at 532 nm. The size distribution was determined from the vesicle diffusion coefficients by standard analysis [52]. Thirty μg of protein for each sample, exosomes, and cells, were analyzed by western blot for Alix (3A9-Cell Signaling Technology #2171S),) Tsg101 (Santa Cruz Biotechnology sc-7964) and HSC70 (Santa Cruz Biotechnology sc-7298).

Uptake of exosomes by HUVECs

Exosomes from Huh7, CD90+ Huh7 and SkHep cells were labeled with PKH26 according to supplier's instructions, suspended in low serum medium (5 μg/ml), and incubated with HUVECs for 1, 3, and 6 hours at 4° or 37°C. After incubation, cells were processed as previously described [26].

HUVECs treatment

HUVECs were grown at a density of 100.000cells/well in a 12 wells plate, and treated for 18 hours with 5 μg/ml of exosomes in low serum medium; untreated cells were considered control. Plasmid for psiCHECK2-H19 and the Empty vector psiCHECK2 (kindly provided by Dr Y. Huang [45]]), H19 siRNA(SR319206B Origene Technologies) and scramble negative control (SR30004 Origene Technologies) were transfected in HUVECs with Attractene Transfection Reagent (cat.number.1051531, Quiagen) following manufacturer's indications.

RNA extraction and real-time PCR

RNA was extracted using the commercially available illustra RNAspin Mini Isolation Kit (GE Healthcare), according to manufacturer's instructions. Total RNA was reverse-transcribed to cDNA using the High Capacity cDNA Reverse Transcription Kit (Applied Biosystem). RT-QPCR was done in 48-well plates using the Step-One Real-Time PCR system (Applied Biosystem). Real-time PCR was performed in duplicates for each data point. For sybr-green method the oligonucleotide used were *β-actin* for5'-ATCAAGATCATTGCTCCTCCTGA-3'rev 5'CTGCTTGCTGATCCACATCTG-3'; H19 for5'-GCACCTTGGACATCTGGAGT-3'rev5'-TTCTTTCCAGCCCTAGCTCA-3', *VEGF* for5'-CGAGGGCCTGGAGTGTGT-3'rev5'-CGCATAATCTGCATGGTGATG-3', *VEGF-R1* for5'-CGGTCAACAAAGTCGGGAGA-3'rev5'-CAGTGCACCACAAAGACACG-3', *VE-CADHERIN* for5'-GATCAAGTCAAGCGTGAGTCG-3' rev5'-AGCCTCTCAATGGCGAACAC-3'. *VCAM1*, ICAM, H19 and *β-actin* transcript levels were measured by TaqMan Real-Time PCR using the TaqMan gene expression assay: Hs00174239_m1, HS 00277001_m1, Hs00262142_g1 and Hs99999903_m1, respectively (Life Technologies,). Changes in the target mRNA content relative to housekeeping were determined with the ΔΔct Method.

Endothelial tube formation assay

HUVECs were seeded at 50,000 cells/well in growth factor-reduced Matrigel-coated 24 well plate and incubated up to 2 hours at 37°C. Tube formation was examined under an inverted microscope and photographed at 20× magnification. The length of the cables was measured manually with IMAGE-J software (http://rsbweb.nih.gov/ij/).

FACS analyses

Two hundred thousand (200,000) cells were washed in PBS and incubated with 0.5 µg ICAM-1-FITC (sc-107, Santa Cruz). Viable cells were gated by forward and side scatter, and analyzed on 100,000 acquired events for each sample. Samples were analyzed on a Partec CyFlow Space using the Partec FloMax® software.

Adhesion assay

In order to evaluate the ability of CD90+ Huh7 cells and SkHep cells to adhere to HUVECs, an adhesion assay was performed, as previously described [26].

ELISA

HUVEC conditioned medium was collected 18 hours after exosome treatment or transfection with pH19 or pEmpty. VEGF concentrations were quantified using the ELISA kit (KHG0111, LifeTechnologies), according to manufacturer's protocol.

Array for long non-coding RNA

In order to study lncRNA expressed in the sorted population, a LncProfiler lncRNA qPCR array was performed (System Bioscience) on Huh7, CD90+Huh7 cells and their exosomes following manufacturer's indications. After amplification, ΔΔct of CD90+Huh7 was normalized on ΔΔct of

Huh7, and data were expressed as fold induction of the sorted population compared with the parental cells.

Statistical analysis

In vitro experiments were repeated three times, giving reproducible results. Data are presented as mean values ± standard deviation (SD) of three independent experiments. Statistical analysis was done using Prism 4 (GraphPad Software Inc., San Diego, CA, USA); one-way ANOVA (non-parametric) was performed, followed by Dunnett's multiple comparison test.

Acknowledgments: MIUR Ministero dell'Universita e Ricerca Scientifica (FIRB 2012-RBFR12NSCF_002); AIRC Associazione Italiana per la Ricerca sul Cancro (12763)

References:

1. Forner A, Llovet JM, Bruix J. Hepatocellular carcinoma. Lancet. 2012;379(9822):1245-55. doi:10.1016/S0140-6736(11)61347-0S0140-6736(11)61347-0 [pii].

2. Uchino K, Tateishi R, Shiina S, Kanda M, Masuzaki R, Kondo Y et al. Hepatocellular carcinoma with extrahepatic metastasis: clinical features and prognostic factors. Cancer. 2011;117(19):4475-83. doi:10.1002/cncr.25960.

3. Yoo DJ, Kim KM, Jin YJ, Shim JH, Ko GY, Yoon HK et al. Clinical outcome of 251 patients with extrahepatic metastasis at initial diagnosis of hepatocellular carcinoma: does transarterial chemoembolization improve survival in these patients? Journal of gastroenterology and hepatology. 2011;26(1):145-54. doi:10.1111/j.1440-1746.2010.06341.x.

4. Roncalli M, Park YN, Di Tommaso L. Histopathological classification of hepatocellular carcinoma. Digestive and liver disease : official journal of the Italian Society of Gastroenterology and the Italian Association for the Study of the Liver. 2010;42 Suppl 3:S228-34. doi:10.1016/S1590-8658(10)60510-5.

5. Suzuki A, Zheng Y, Kondo R, Kusakabe M, Takada Y, Fukao K et al. Flow-cytometric separation and enrichment of hepatic progenitor cells in the developing mouse liver. Hepatology. 2000;32(6):1230-9. doi:10.1053/jhep.2000.20349.

6. Oishi N, Yamashita T, Kaneko S. Molecular biology of liver cancer stem cells. Liver cancer. 2014;3(2):71-84. doi:10.1159/000343863.
7. Yoon SK. The biology of cancer stem cells and its clinical implication in hepatocellular carcinoma. Gut and liver. 2012;6(1):29-40. doi:10.5009/gnl.2012.6.1.29.
8. Yao Z, Mishra L. Cancer stem cells and hepatocellular carcinoma. Cancer biology & therapy. 2009;8(18):1691-8.
9. Chiba T, Kita K, Zheng YW, Yokosuka O, Saisho H, Iwama A et al. Side population purified from hepatocellular carcinoma cells harbors cancer stem cell-like properties. Hepatology. 2006;44(1):240-51. doi:10.1002/hep.21227.
10. Ma S, Tang KH, Chan YP, Lee TK, Kwan PS, Castilho A et al. miR-130b Promotes CD133(+) liver tumor-initiating cell growth and self-renewal via tumor protein 53-induced nuclear protein 1. Cell stem cell. 2010;7(6):694-707. doi:10.1016/j.stem.2010.11.010.
11. Yang ZF, Ho DW, Ng MN, Lau CK, Yu WC, Ngai P et al. Significance of CD90+ cancer stem cells in human liver cancer. Cancer cell. 2008;13(2):153-66. doi:10.1016/j.ccr.2008.01.013.
12. Yamashita T, Honda M, Nakamoto Y, Baba M, Nio K, Hara Y et al. Discrete nature of EpCAM+ and CD90+ cancer stem cells in human hepatocellular carcinoma. Hepatology. 2013;57(4):1484-97. doi:10.1002/hep.26168.
13. Rege TA, Hagood JS. Thy-1, a versatile modulator of signaling affecting cellular adhesion, proliferation, survival, and cytokine/growth factor responses. Biochimica et biophysica acta. 2006;1763(10):991-9. doi:10.1016/j.bbamcr.2006.08.008.
14. Herrera MB, Bruno S, Buttiglieri S, Tetta C, Gatti S, Deregibus MC et al. Isolation and characterization of a stem cell population from adult human liver. Stem cells. 2006;24(12):2840-50. doi:10.1634/stemcells.2006-0114.
15. Sukowati CH, Anfuso B, Torre G, Francalanci P, Croce LS, Tiribelli C. The expression of CD90/Thy-1 in hepatocellular carcinoma: an in vivo and in vitro study. PloS one. 2013;8(10):e76830. doi:10.1371/journal.pone.0076830.
16. Lingala S, Cui YY, Chen X, Ruebner BH, Qian XF, Zern MA et al. Immunohistochemical staining of cancer stem cell markers in hepatocellular carcinoma. Experimental and molecular pathology. 2010;89(1):27-35. doi:10.1016/j.yexmp.2010.05.005.
17. Yu XH, Xu LB, Liu C, Zhang R, Wang J. Clinicopathological characteristics of 20 cases of hepatocellular carcinoma with bile duct tumor thrombi. Digestive diseases and sciences. 2011;56(1):252-9. doi:10.1007/s10620-010-1256-8.
18. Lu JW, Chang JG, Yeh KT, Chen RM, Tsai JJ, Hu RM. Overexpression of Thy1/CD90 in human hepatocellular carcinoma is associated with HBV infection and poor prognosis. Acta histochemica. 2011;113(8):833-8. doi:10.1016/j.acthis.2011.01.001.
19. Yang ZF, Ngai P, Ho DW, Yu WC, Ng MN, Lau CK et al. Identification of local and circulating cancer stem cells in human liver cancer. Hepatology. 2008;47(3):919-28. doi:10.1002/hep.22082.
20. Guo Z, Li LQ, Jiang JH, Ou C, Zeng LX, Xiang BD. Cancer stem cell markers correlate with early recurrence and survival in hepatocellular carcinoma. World J Gastroenterol. 2014;20(8):2098-106. doi:10.3748/wjg.v20.i8.2098.
21. Fang L, Zhang HB, Li H, Fu Y, Yang GS. miR-548c-5p inhibits proliferation and migration and promotes apoptosis in CD90(+) HepG2 cells. Radiology and oncology. 2012;46(3):233-41. doi:10.2478/v10019-012-0025-z.
22. Fontana S, Saieva L, Taverna S, Alessandro R. Contribution of proteomics to understanding the role of tumor-derived exosomes in cancer progression: state of the art and new perspectives. Proteomics. 2013;13(10-11):1581-94. doi:10.1002/pmic.201200398.

23. Thery C, Amigorena S, Raposo G, Clayton A. Isolation and characterization of exosomes from cell culture supernatants and biological fluids. Current protocols in cell biology / editorial board, Juan S Bonifacino [et al]. 2006;Chapter 3:Unit 3 22. doi:10.1002/0471143030.cb0322s30.
24. Braicu C, Tomuleasa C, Monroig P, Cucuianu A, Berindan-Neagoe I, Calin GA. Exosomes as divine messengers: are they the Hermes of modern molecular oncology? Cell death and differentiation. 2015;22(1):34-45. doi:10.1038/cdd.2014.130.
25. Kogure T, Yan IK, Lin WL, Patel T. Extracellular Vesicle-Mediated Transfer of a Novel Long Noncoding RNA TUC339: A Mechanism of Intercellular Signaling in Human Hepatocellular Cancer. Genes & cancer. 2013;4(7-8):261-72. doi:10.1177/1947601913499020.
26. Taverna S, Flugy A, Saieva L, Kohn EC, Santoro A, Meraviglia S et al. Role of exosomes released by chronic myelogenous leukemia cells in angiogenesis. Int J Cancer. 2012;130(9):2033-43. doi:10.1002/ijc.26217.
27. Corrado C, Raimondo S, Saieva L, Flugy AM, De Leo G, Alessandro R. Exosome-mediated crosstalk between chronic myelogenous leukemia cells and human bone marrow stromal cells triggers an interleukin 8-dependent survival of leukemia cells. Cancer letters. 2014;348(1-2):71-6. doi:10.1016/j.canlet.2014.03.009

S0304-3835(14)00154-2 [pii].
28. Webber J, Yeung V, Clayton A. Extracellular vesicles as modulators of the cancer microenvironment. Semin Cell Dev Biol. 2015;40:27-34. doi:S1084-9521(15)00025-7 [pii]

10.1016/j.semcdb.2015.01.013.
29. Eun JR, Jung YJ, Zhang Y, Zhang Y, Tschudy-Seney B, Ramsamooj R et al. Hepatoma SK Hep-1 cells exhibit characteristics of oncogenic mesenchymal stem cells with highly metastatic capacity. PloS one. 2014;9(10):e110744. doi:10.1371/journal.pone.0110744.
30. Yu FJ, Zheng JJ, Dong PH, Fan XM. Long non-coding RNAs and hepatocellular carcinoma. Molecular and clinical oncology. 2015;3(1):13-7. doi:10.3892/mco.2014.429.
31. Sun J, Bie B, Zhang S, Yang J, Li Z. Long Non-Coding RNAs: Critical Players in Hepatocellular Carcinoma. International journal of molecular sciences. 2014;15(11):20434-48. doi:10.3390/ijms151120434.
32. Pan YF, Qin T, Feng L, Yu ZJ. Expression profile of altered long non-coding RNAs in patients with HBV-associated hepatocellular carcinoma. Journal of Huazhong University of Science and Technology Medical sciences = Hua zhong ke ji da xue xue bao Yi xue Ying De wen ban = Huazhong keji daxue xuebao Yixue Yingdewen ban. 2013;33(1):96-101. doi:10.1007/s11596-013-1078-y.
33. Yang X, Xie X, Xiao YF, Xie R, Hu CJ, Tang B et al. The emergence of long non-coding RNAs in the tumorigenesis of hepatocellular carcinoma. Cancer letters. 2015. doi:S0304-3835(15)00139-1 [pii]

10.1016/j.canlet.2015.02.035.
34. George J, Patel T. Noncoding RNA as therapeutic targets for hepatocellular carcinoma. Semin Liver Dis. 2015;35(1):63-74. doi:10.1055/s-0034-1397350.
35. Ma C, Nong K, Zhu H, Wang W, Huang X, Yuan Z et al. H19 promotes pancreatic cancer metastasis by derepressing let-7's suppression on its target HMGA2-mediated EMT. Tumour biology : the journal of the International Society for Oncodevelopmental Biology and Medicine. 2014;35(9):9163-9. doi:10.1007/s13277-014-2185-5.
36. Matouk IJ, Raveh E, Abu-lail R, Mezan S, Gilon M, Gershtain E et al. Oncofetal H19 RNA promotes tumor metastasis. Biochimica et biophysica acta. 2014;1843(7):1414-26. doi:10.1016/j.bbamcr.2014.03.023.
37. Takahashi K, Yan IK, Kogure T, Haga H, Patel T. Extracellular vesicle-mediated transfer of long non-coding RNA ROR modulates chemosensitivity in human hepatocellular cancer. FEBS open bio. 2014;4:458-67. doi:10.1016/j.fob.2014.04.007.

38. Ye J, Wu D, Wu P, Chen Z, Huang J. The cancer stem cell niche: cross talk between cancer stem cells and their microenvironment. Tumour biology : the journal of the International Society for Oncodevelopmental Biology and Medicine. 2014;35(5):3945-51. doi:10.1007/s13277-013-1561-x.
39. Kahlert C, Kalluri R. Exosomes in tumor microenvironment influence cancer progression and metastasis. J Mol Med (Berl). 2013;91(4):431-7. doi:10.1007/s00109-013-1020-6.
40. Takahashi K, Yan IK, Haga H, Patel T. Modulation of hypoxia-signaling pathways by extracellular linc-RoR. J Cell Sci. 2014;127(Pt 7):1585-94. doi:10.1242/jcs.141069

jcs.141069 [pii].
41. Moran VA, Perera RJ, Khalil AM. Emerging functional and mechanistic paradigms of mammalian long non-coding RNAs. Nucleic acids research. 2012;40(14):6391-400. doi:10.1093/nar/gks296.
42. Zhang Y, Tycko B. Monoallelic expression of the human H19 gene. Nature genetics. 1992;1(1):40-4. doi:10.1038/ng0492-40.
43. Hark AT, Schoenherr CJ, Katz DJ, Ingram RS, Levorse JM, Tilghman SM. CTCF mediates methylation-sensitive enhancer-blocking activity at the H19/Igf2 locus. Nature. 2000;405(6785):486-9. doi:10.1038/35013106.
44. Dey BK, Pfeifer K, Dutta A. The H19 long noncoding RNA gives rise to microRNAs miR-675-3p and miR-675-5p to promote skeletal muscle differentiation and regeneration. Genes Dev. 2014;28(5):491-501. doi:10.1101/gad.234419.113

gad.234419.113 [pii].
45. Kallen AN, Zhou XB, Xu J, Qiao C, Ma J, Yan L et al. The imprinted H19 lncRNA antagonizes let-7 microRNAs. Mol Cell. 2013;52(1):101-12. doi:10.1016/j.molcel.2013.08.027

S1097-2765(13)00627-8 [pii].
46. Yang F, Bi J, Xue X, Zheng L, Zhi K, Hua J et al. Up-regulated long non-coding RNA H19 contributes to proliferation of gastric cancer cells. FEBS J. 2012;279(17):3159-65. doi:10.1111/j.1742-4658.2012.08694.x.
47. Luo M, Li Z, Wang W, Zeng Y, Liu Z, Qiu J. Long non-coding RNA H19 increases bladder cancer metastasis by associating with EZH2 and inhibiting E-cadherin expression. Cancer letters. 2013;333(2):213-21. doi:10.1016/j.canlet.2013.01.033.
48. Matouk IJ, DeGroot N, Mezan S, Ayesh S, Abu-lail R, Hochberg A et al. The H19 non-coding RNA is essential for human tumor growth. PloS one. 2007;2(9):e845. doi:10.1371/journal.pone.0000845.
49. Fellig Y, Ariel I, Ohana P, Schachter P, Sinelnikov I, Birman T et al. H19 expression in hepatic metastases from a range of human carcinomas. Journal of clinical pathology. 2005;58(10):1064-8. doi:10.1136/jcp.2004.023648.
50. Kim DK, Zhang L, Dzau VJ, Pratt RE. H19, a developmentally regulated gene, is reexpressed in rat vascular smooth muscle cells after injury. J Clin Invest. 1994;93(1):355-60. doi:10.1172/JCI116967.
51. Han DK, Khaing ZZ, Pollock RA, Haudenschild CC, Liau G. H19, a marker of developmental transition, is reexpressed in human atherosclerotic plaques and is regulated by the insulin family of growth factors in cultured rabbit smooth muscle cells. J Clin Invest. 1996;97(5):1276-85. doi:10.1172/JCI118543.
52. Noto R, Santangelo MG, Ricagno S, Mangione MR, Levantino M, Pezzullo M et al. The tempered polymerization of human neuroserpin. PloS one. 2012;7(3):e32444. doi:10.1371/journal.pone.0032444

PONE-D-11-20702 [pii].

Figure Legends

Fig. 1: CD90+ population. A. Huh7 and sorted CD90+ Huh7 were stained for hepatocytic (HF4alpha), epithelial (E-Cadherin) and mesenchymal (Vimentin) markers, in blue the nuclear staining with DAPI. **Characterization of isolated exosomes**. **B.** Dynamic light scattering of vesicles isolated from Huh7 (in black) and from CD90+Huh7 cells (in red). **C.** Western blot for endosomal markers Alix,Tsg101 and HSC70 in Huh7 and 90+ population with their relative exosomes. **D.** Confocal microscopy analysis on HUVECs treated for 1, 3, and 6 hours with 5μg/ml of exosomes from CD90+ or Huh7 cells. HUVECs were stained with phalloidin Alexa Fluor488 (green), nuclear counterstaining was done using DAPI (blue), exosomes were labelled with PKH26 (red).

Fig. 2: HUVECs characterization after exosome treatment: A. RT-PCR analyses for VEGF, VEGF-R and ICAM1 were done on HUVECs 18 hours after treatment with CD90+ or Huh7-derived exosomes (5μg/ml). ΔΔct expressed as fold of induction (FOI) compared with control (untreated cells). ***$p<0.001$; *$p<0.05$.**B left panel**. ELISA for VEGF released by HUVECs 18 hours after treatment with CD90+exo or Huh7exo. Untreated cells were used as control. *$p<0.01$. **B middle-right panels:** Tubulogenesis analysis. Phase contrast micrographs (20x) and quantification of matrigel assay expressed as length of cable as arbitrary unit. **C.** FACS analysis for ICAM-1 on HUVECs 18 hours after treatment with Huh7exo or CD90+exo, respectively. **D** Adhesion capacity. **Left panel:** Phase contrast micrographs (20x) showing the adhesion of CD90+cells on HUVEC monolayer pre-treated with Huh7exo or CD90+exo. **Right panel:** Quantification of adhesion established by counting the number of adherent CD90+cells (violet) per field; **$p<0.01$

Fig3 A. LncRNAs expressed in CD90+ cells and their exosomes (left and middle panel). Data are expressed as fold induction compared with Huh7 mix population. Of the 90 lncRNAs analyzed, only

those over-expressed more than ten-fold in CD90+ cells were considered. Listed on the right the lncRNA up-regulated in HCC. **Right panel:** LncRNA Profile in exosomes released by CD90+Huh7. Data are expressed as fold of induction compared with exosomes from Huh7 parental cells. **B. H19 analysis.** Real-time PCR analysis for H19 expression in exosomes derived from Huh7 or CD90+ cells. Exosomes were treated with RNase and subsequently processed for RNA extraction and retrotrascription. Data were normalized for β-actin and ΔΔct indicated as fold of induction compared with Huh7-derived exosomes. **p<0.01. **C**. Real-time PCR for H19 on HUVEC 18 hours after treatment with CD90+exo or Huh7exo. Data were normalized for β-actin and ΔΔct indicated as fold of induction compared with control (untreated cells). ***p<0.001

Fig. 4: H19 overexpression. A. Right panel: Real-time PCR performed on HUVECs 18 hours post-transfection. Data were normalized for β-actin and ΔΔct expressed as fold of induction pH19 vs. pEmpty **p<0.01; * p<0.05. **Left panel**: ELISA assay for VEGF level in supernatant from HUVECs 18 hours after transfection. ***p<0.001. **B.** Phase contrast (20x) of tubulogenesis assay performed 18 hours after transfection. Left panel: quantification of matrigel assay expressed as length of cable as arbitrary unit. **C.** FACS analysis for ICAM expression in HUVEC transfected cells. **D.** Phase contrast micrographs (20x) showing the adhesion of CD90+cells on HUVEC monolayer transfected with pEmpty or pH19. Quantification was calculated by counting the number of adherent CD90+ cells (violet) per field. **p<0.01

Fig. 5: Real-time PCR for H19, VEGF, VEGFR1 and ICAM1 from HUVECs transfected with H19 siRNA or negative scramble and treated with CD90+exo. Data were normalized for β-actin and ΔΔct expressed as fold of induction siRNA H19 vs. negative control. **p<0.01, ***p<0.001 **B.** ELISA assay for VEGF detection on the supernatant from HUVECs treated as indicated above. ***p<0.001.

Figure 1

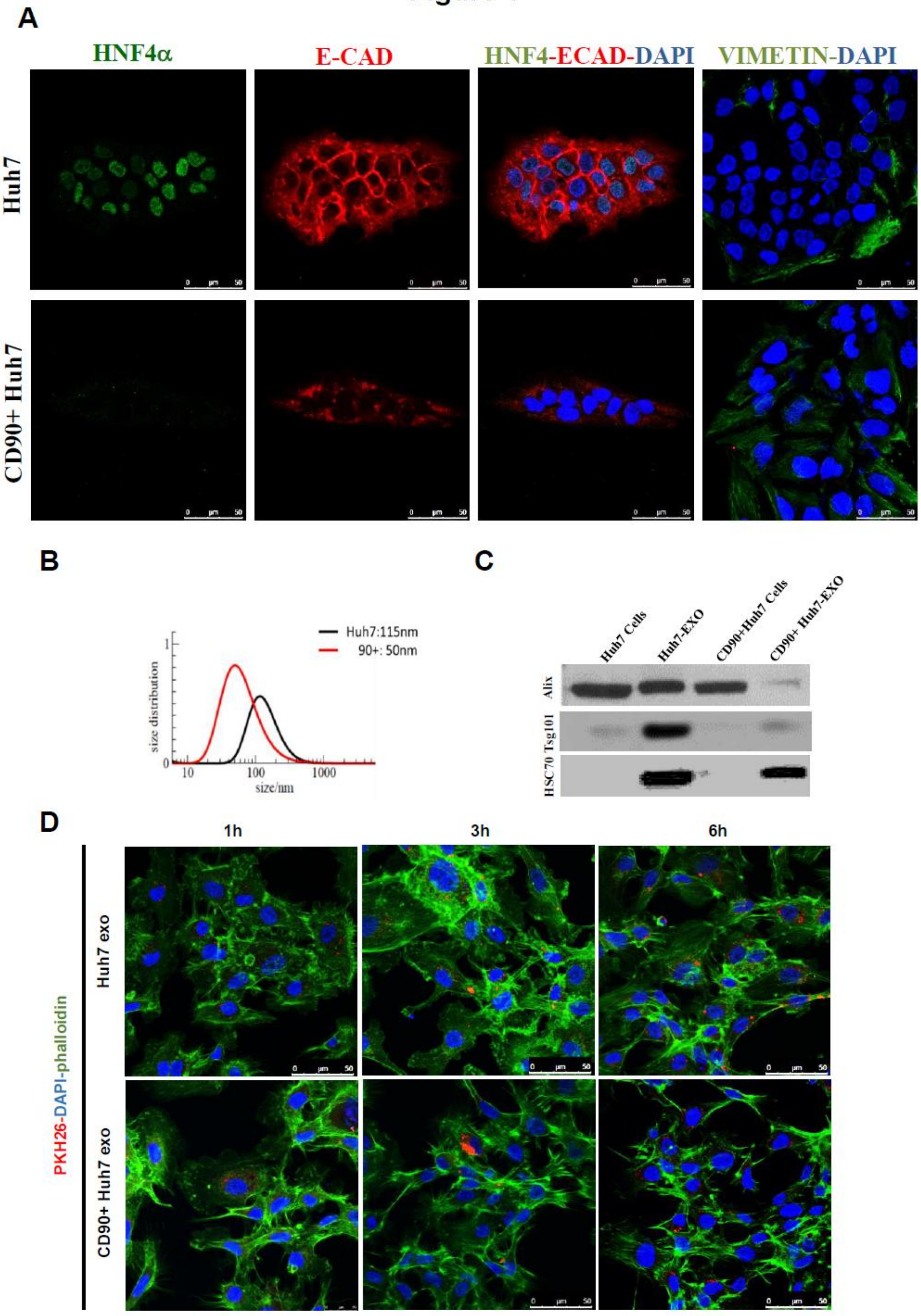

A
HNF4α
E-CAD
HNF4-ECAD-DAPI
VIMETIN-DAPI
Huh7
CD90+ Huh7
B
Huh7:115nm
90+: 50nm
size distribution
size/nm
C
Huh7 Cells
Huh7-EXO
CD90+Huh7 Cells
CD90+ Huh7-EXO
Alix
Tsg101
HSC70
D
1h
3h
6h
Huh7 exo
CD90+ Huh7 exo
PKH26-DAPI-phalloidin

Figure 2

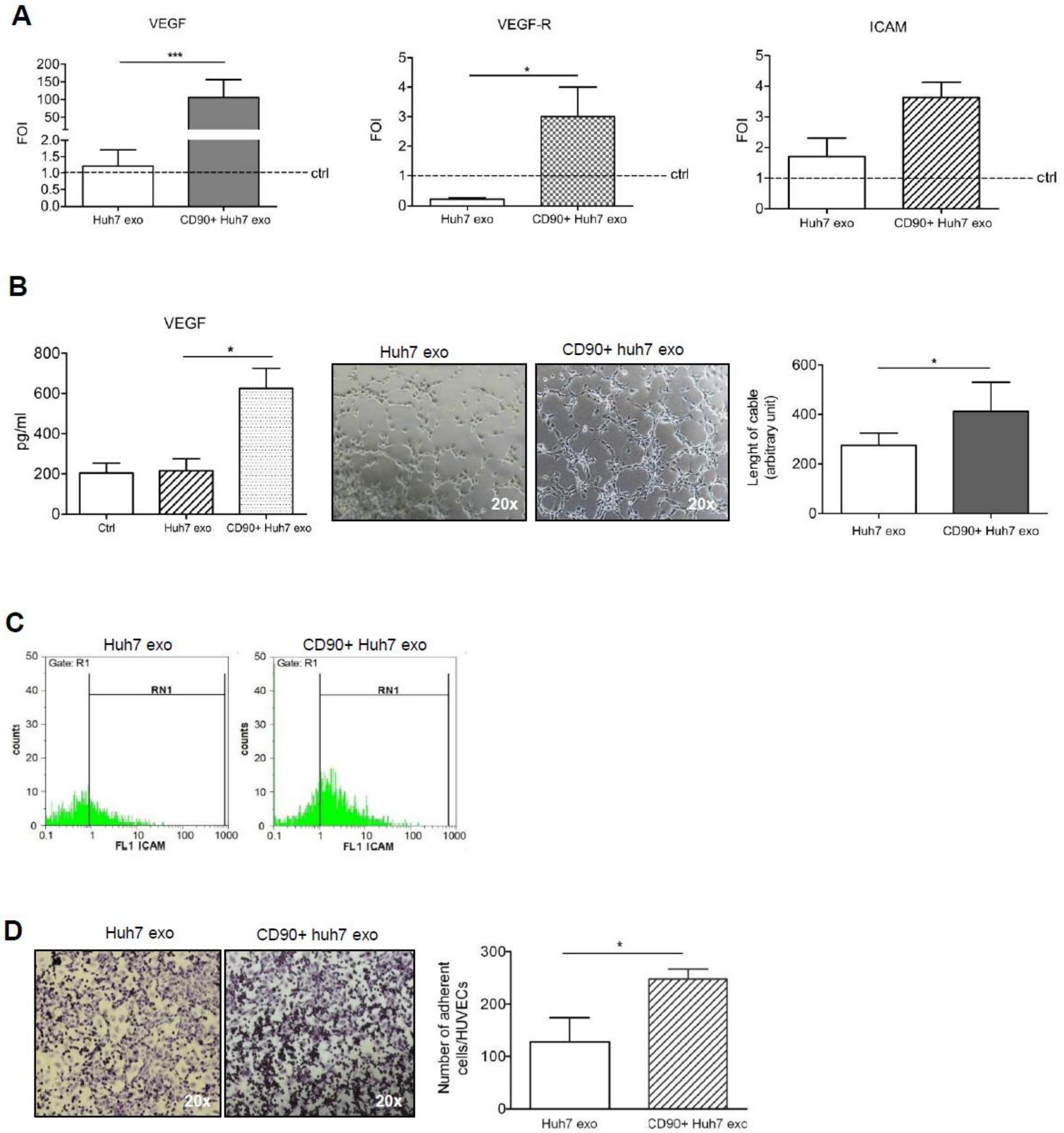
A
VEGF
FOI
ctrl
Huh7 exo
CD90+ Huh7 exo
VEGF-R
ICAM
B
VEGF
pg/ml
Ctrl
Huh7 exo
CD90+ huh7 exo
20x
Lenght of cable (arbitrary unit)
C
Huh7 exo
CD90+ Huh7 exo
Gate: R1
RN1
counts
FL1 ICAM
D
Huh7 exo
CD90+ huh7 exo
20x
Number of adherent cells/HUVECs

Figure 3

A

LncRNA	Fold
21A	119
Air	1846
Alpha 280	294
Alpha 250	80
ANRIL	121
anti-NOS2A	2469
antiPeg11	310
BC200	70
DISC2 (family)	325
DLG2AS (family)	1331
E2F4 antisense	17988
EGO B	18
Emx2os	18
H19	18
H19 antisense	282
H19 upstream conserv	222
HAR1B	19
HOTAIR	1289
HOTAIRM1	159
Hoxa11as	280
HOXA6as	18
HULC	18
IGF2AS (family)	147
KRASP1	145
L1PA16	141
lincRNA-RoR	221

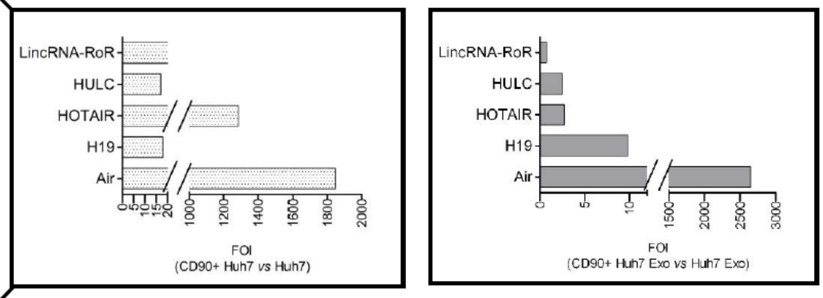

B

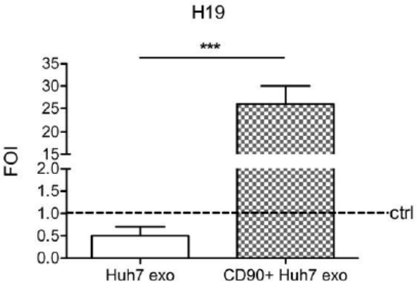

C

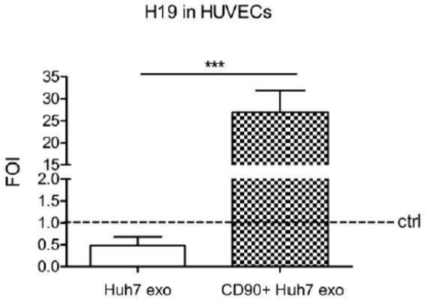

Figure 4

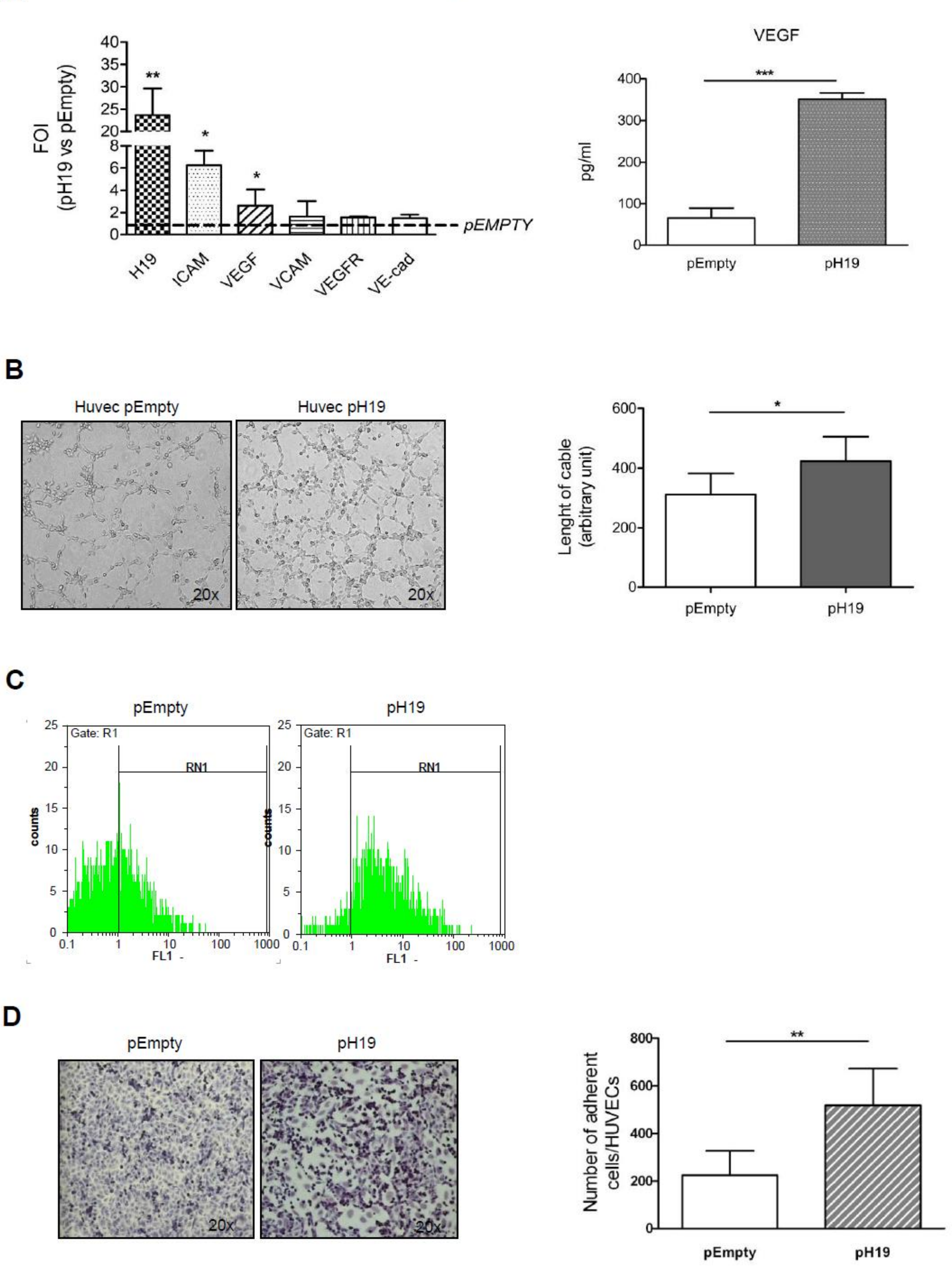
A
FOI
(pH19 vs pEmpty)
40
35
30
25
20
8
6
4
2
0
**
*
*
pEMPTY
H19
ICAM
VEGF
VCAM
VEGFR
VE-cad
VEGF
pg/ml
400
300
200
100
0
***
pEmpty
pH19
B
Huvec pEmpty
Huvec pH19
20x
20x
Lenght of cable
(arbitrary unit)
600
400
200
0
*
pEmpty
pH19
C
pEmpty
pH19
Gate: R1
RN1
counts
25
20
15
10
5
0
0.1
1
10
100
1000
FL1 -
D
pEmpty
pH19
20x
20x
Number of adherent
cells/HUVECs
800
600
400
200
0
**
pEmpty
pH19

Figure 5

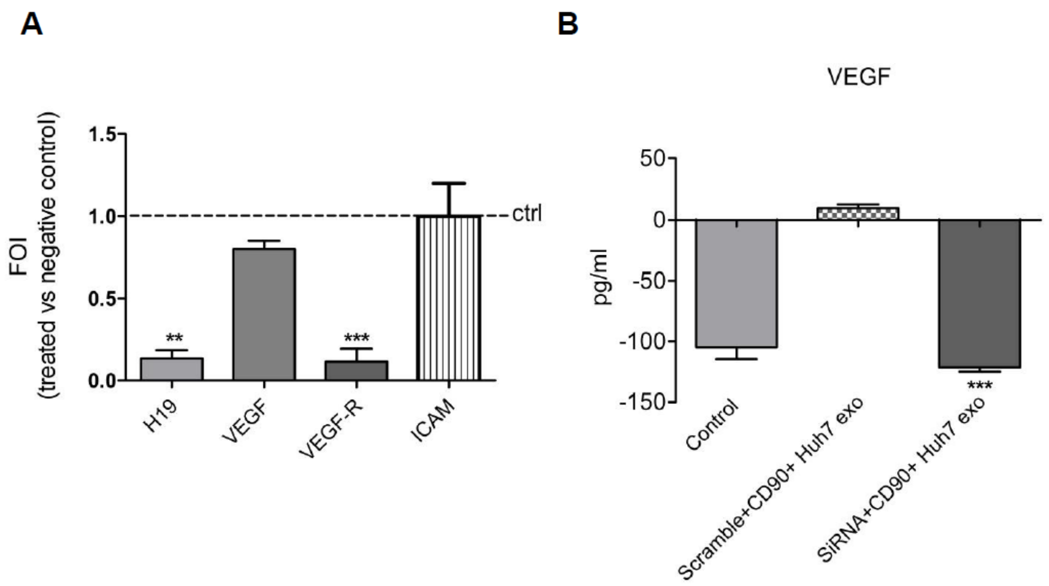
A
B
FOI
(treated vs negative control)
1.5
1.0
0.5
0.0
ctrl
**
***
H19
VEGF
VEGF-R
ICAM
VEGF
pg/ml
50
0
-50
-100
-150
***
Control
Scramble+CD90+ Huh7 exo
SiRNA+CD90+ Huh7 exo
